# An ultra-high-performance liquid chromatography tandem mass spectrometry method for oxidative stress biomarker analysis in wastewater

**DOI:** 10.1007/s00216-019-01667-8

**Published:** 2019-02-23

**Authors:** Natalie Sims, Jack Rice, Barbara Kasprzyk-Hordern

**Affiliations:** 1 Department of Chemistry, University of Bath, Bath, BA2 7AY UK; 2 Centre for Doctoral Training in Sustainable Chemical Technologies, University of Bath, Bath, BA2 7AY UK

**Keywords:** Oxidative stress, Biomarkers, Wastewater, Urban water fingerprinting

## Abstract

Reported herein is the development of an analytical method for the detection of four oxidative stress biomarkers in wastewater using ultra-high-performance liquid chromatography coupled with tandem mass spectrometry (UHPLC-MS/MS) and solid phase extraction (SPE). The following four biomarkers of oxidative stress and lipid peroxidation have been investigated: hydroxynonenal–mercapturic acid (HNE-MA), 8-*iso*-prostglandin F2beta (8-*iso*-PGF_2β_), 8-nitroguanine (8-NO_2_Gua) and 8-hydroxy-2-deoxyguanosine (8-OHdG). The method showed very good performance: accuracy (> 87%), precision (> 90%), method quantification limits (1.3–3.0 ng L^−1^) and biomarker stability in wastewater in the case of HNE-MA, 8-OHdG and 8-*iso-*PGF_2β_. In contrast, 8-NO_2_Gua was found to be less stable in wastewater, which affected its method performance: accuracy (> 63%), precision (> 91%) and method quantification limits (85.3 ng L^−1^). Application of the developed method resulted in, for the first time, HNE-MA being successfully observed and quantified within wastewater over a study period of a week (displayed average daily loads per capita of 48.9 ± 4.1 mg/1000/people/day). 8-*iso*-PGF_2β_ was detected with good intensity but could not be quantified due to co-elution with other isomers. 8-OHdG was detected, albeit at < MQL. This study demonstrates the potential for expanding on the possible endogenous biomarkers of health used in urban water fingerprinting to aid in measuring health in near-real time on a community-wide scale.

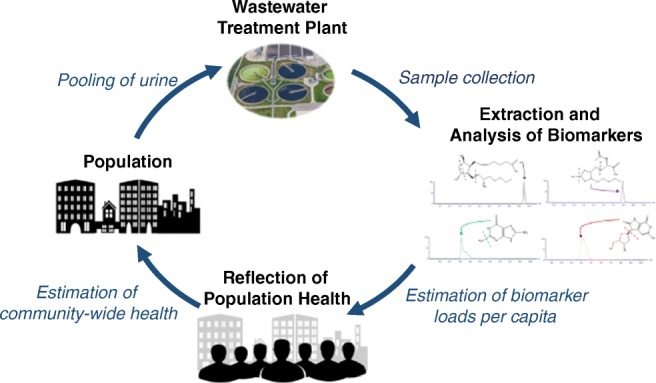

## Introduction

Wastewater-based epidemiology is a rapidly developing and innovative technique that analyses human metabolic excretion products in the wastewater of a defined population [1]. The wastewater of a community is an incredibly valuable, yet traditionally under-estimated, source of knowledge. The analysis of targeted aspects of biological and chemical information wastewater contains can offer a unique reflection of health upon the population that contributes. The concept of WBE has already experienced enormous successes from communities to international scales to evaluate and compare trends in illicit drug usage [2–4], pharmaceuticals [5], alcohol [6–8] and tobacco consumption [9–11]. Recently the potential for WBE to evaluate and monitor community-wide public health has been highlighted by analysing endogenous urinary biomarkers of human health and disease [12–14].

Currently monitoring public health is done via conventional epidemiological studies. These are based upon existing resources including morbidity data, prescription rates and questionnaires [12, 15]. However, the results from such sources of information can be subject to bias and are not always representative of a whole community, hence can give misleading results. One of the crucial disadvantages of current approaches is there is no capacity for real-time monitoring of health on a community scale. This results in difficulties in establishing trends in a population’s health and causes serious issues in implementing appropriate and effective healthcare interventions.

An increasingly popular branch of epidemiology studies based upon the assessment of human exposure to external factors such as environmental pollution is human biomonitoring. This technique involves the detection and analysis of biomarkers of interest in biological samples of individuals. Such samples can include saliva, blood, tissue or excretion products [16]. However, this process is expensive and time-consuming and results in only a small portion of a population being investigated which might not be representative of a population as a whole [17]. Furthermore, such techniques require samples from thousands of patients in a defined geographic location and the selection of a control group for comparison can be challenging.

A possible solution to these drawbacks is to use WBE as complementary tool to conventional public health assessments [14]. The ability to analyse and monitor endogenous biomarkers of disease within the wastewater of a community in near-real time could offer an unbiased reflection of the health of the population that contributes. It has been proposed that the evaluation of oxidative stress biomarkers could give key information upon the health status of a community [12]. Oxidative stress is defined as the imbalance between reactive oxygen species and the ability of the body to counteract with antioxidants [18]. It is a key characteristic of many acute and chronic diseases including stroke [19], heart disease [20], cancers and respiratory infections [21] as well as being an indicator for certain lifestyle factors such as excessive smoking and alcohol consumption [22, 23]. Indications of oxidative stress are often reflected through elevated levels of specific biomarkers within parts of body, including blood plasma, and urine. Higher levels of oxidative stress biomarkers in individuals within populations have not only been linked with various diseases and lifestyle factors but have also been correlated with environmental exposure, for example air pollution [24, 25]. As a result, not only could measurement of cumulative stress give information about the general health of a community but could also give valuable data on the exposure to external factors such as anthropogenic pollution, an area of study where still very little is known.

In particular, a handful of key oxidative stress biomarkers have been well-studied within urine, with various analytical methods developed for 8-*iso*-prostaglandin F2alpha (8-*iso*-PGF_2α_), 8-nitroguaninne (8-NO_2_Gua) and 8-hydroxy-2-deoxyguanosine (8-OHdG) and hydroxynonenal–mercapturic acid (HNE-MA) [26–28]. However, to date, only one biomarker of oxidative stress has been observed and quantified by WBE techniques in wastewater [29]. Wastewater analysis poses many challenges as the matrix itself has significantly higher complexity and interchangeability in comparison to urine. Furthermore, with regard to the biomarkers themselves, the concentrations in wastewater are far lower (sub-ppt levels) than those observed in urine (e.g. ng/mg of creatinine for 8-OHdG [30]). Urinary 8-*iso*-PGF_2α_ is formed within the body from the oxidation of arachidonic acid and is widely-recognised reliable biomarker of oxidative stress with elevated levels typically observed within urine [12, 31–33]. In a unique study by Ryu et al., 8-*iso*-PGF_2α_ was successfully extracted from wastewater samples using highly specific immunoassay approaches [29]. A further study demonstrated 8-*iso*-PGF_2α_ correlated with the major metabolite of smoking in wastewater across 11 cities in Europe [34].

This paper aimed to develop an analytical method using ultra-high-performance liquid chromatography mass spectrometry (UHPLC-MS) to analyse, for the first time, four biomarkers of oxidative stress 8-*iso*-prostglandin F2beta (8-*iso*-PGF_2β_), HNE-MA, 8-NO_2_Gua and 8-OHdG from wastewater through application of solid phase extraction (SPE) techniques. 8-OHdG and 8-NO_2_Gua are reliable markers of oxidative DNA and nitrative DNA damage respectively. Reactive oxygen species (ROS) produced as a result of oxidative stress can not only damage DNA but also cause destruction of the cell membranes in a process known as lipid peroxidation. The urinary biomarker HNE-MA is a key indicator of cell membrane damage and 8-*iso*-PGF_2β_ in an isomer of the reliable oxidative stress marker 8-*iso*-PGF_2α_.

## Materials and methods

### Materials

A total of four biomarkers were selected for method development due to their acknowledged indication of oxidative stress within urine [28]. The standard 8-OHdG was bought from Sigma–Aldrich (UK), its respective internal standard ^15^N_5_-8-OHdG along with 8-NO_2_Gua were purchased from Santa Cruz Biotechnologies (UK). The standards 8-*iso*-PGF_2β_, HNE-MA and the internal standard HNE-MA-d3 were bought from Cayman Chemicals (US). Stock solutions of selected biomarkers were made up by dissolving solid samples in MeOH and all stock solutions were kept in the dark at − 80 °C. Working solutions were diluted from the stock solutions to make up the desired concentrations in 80:20 H_2_O:MeOH. Solvents such as MeOH and toluene were HPLC grade and purchased from Sigma–Aldrich. To remove the risk of basic functional groups reacting with silanols on glass surfaces, all glassware was deactivated using 5% dimethylchlorosilane (DMDCS) in toluene. The silanisation of glass occurred by rinsing with DMDCS before washing twice with toluene and three times with MeOH.

### Solid-phase extraction

**Fig. 1:**
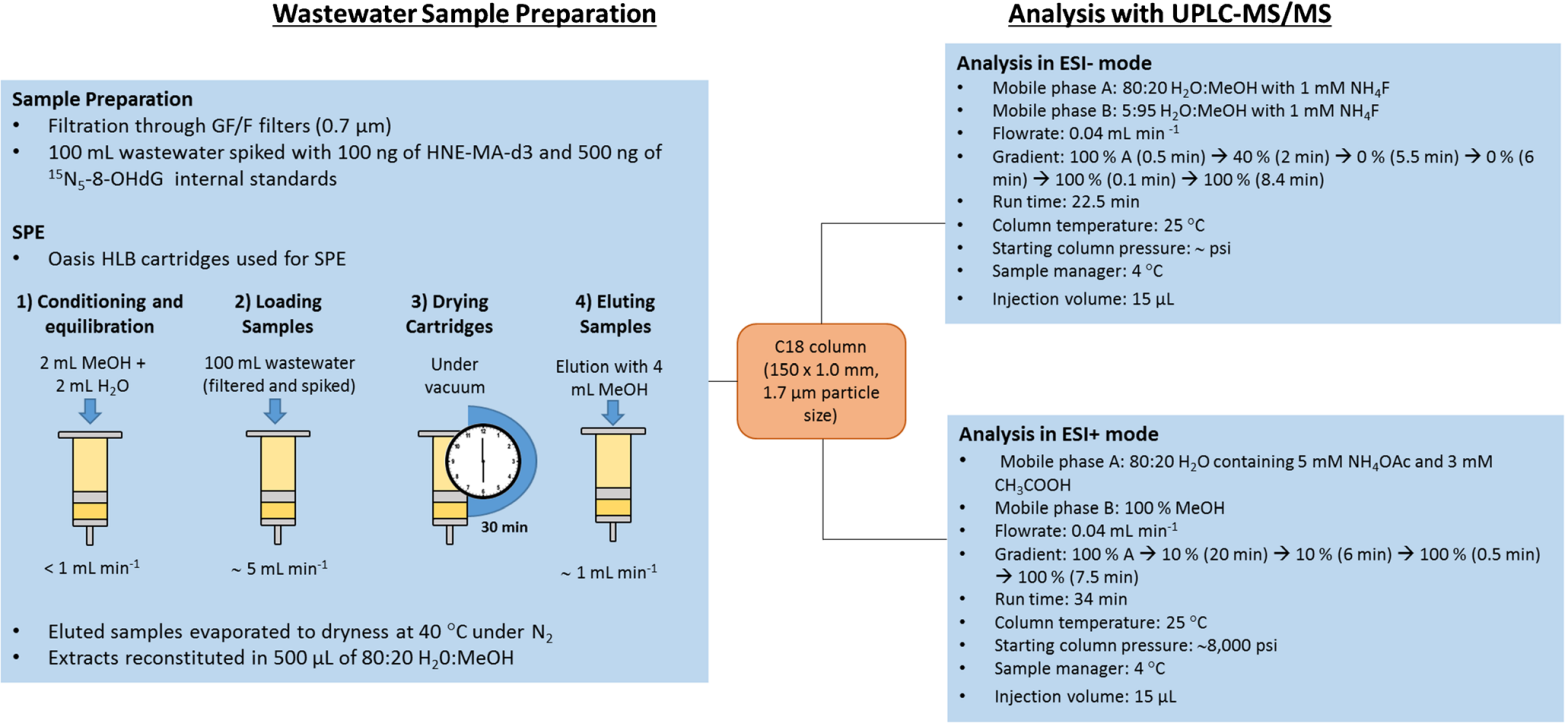
Summary of the wastewater sample preparation and extraction followed by analytical method details

The solid-phase extraction (SPE) procedure followed the protocol previously published by Petrie et al. [35]. For all extractions, Oasis HLB (Waters, hydrophilic-lipophilic-balanced) cartridges (60 mg, 3 mL) were conditioned with 2 mL of MeOH followed by 2 mL of deionised water (pH 7.5) for equilibration. Influent wastewater was aliquoted into 100 mL samples before spiking with internal standard solutions (final concentrations of 100 ng and 500 ng for HNE-MA-d3 and ^15^N_5_-8-OHdG respectively). Spiked influent wastewater samples were then filtered through GF/F filters before loading onto the pre-conditioned HLB cartridges at a flow rate of < 1 mL min^−1^. Once loaded, cartridges were left to dry under vacuum for 30 min. Elution of analytes occurred using 4 mL of MeOH at a steady flow rate of 1 mL min^−1^. Once eluted, samples were evaporated till dry under N_2_, 40 °C using TurboVap evaporator (Calliper, UK) this was then followed by reconstitution with 500 μL of 80:20 H_2_O: MeOH. Samples were transferred to polypropylene vials and 20 μL of sample were injected into the Waters Acquity UPLC system. A graphical representation of the extraction procedures and analytical methodology has been detailed (Fig. 1).

### Liquid chromatography coupled with tandem mass spectrometry

Liquid chromatography was performed using a Waters Acquity UPLC system which was coupled to the Xevo TQD Triple Quadrupole Mass Spectrometer (Waters, UK). Due to ionisation preference of the chosen biomarkers, two methods have been developed for this study. 8-OHdG ionised in ESI positive mode whereas HNE-MA, 8-*iso*-PGF_2β_ and 8-NO_2_Gua ionised preferentially in ESI negative mode (Table 1). Both methods used a reversed-phase BEH C18 column (150 × 1.0 mm, 1.7 μm particle size) (Waters, UK) with a 0.2 μm, 2.1 mm in-line column filter (Water, UK) maintained at 25 °C. Mobile phase used in ESI negative was as follows: A; 80:20 H_2_O:MeOH with 1 mM NH4F (mobile phase A) and 5:95 H_2_O:MeOH with 1 mM NH_4_F (mobile phase B) with the following gradient, 100%A (0.5 min)–40% (2 min)–0%A (5.5 min). Mobile phase used in ESI positive was as follows: A; 80:20 H_2_O:MeOH with 5 mM NH_4_OAc and 0.3% CH_3_COOH (mobile phase A) and MeOH (mobile phase B) with the following gradient, 100%A reduced to 10% over 20 min. The mobile phase flow rate was kept constant at 0.04 mL min^−1^ and a 20 μL injection volume was used in both methods.

**Table 1:** Target biomarkers with MS parameter details and fragment details plus internal standards used

Compound/internal standard	Biomarker	MRM mass transition (*m*/*z*)	Cone voltage (*v*)	Collision energy (*v*)	ESI
8-OHdG ^15^N_5_-8-OHdG	Oxidative DNA damage	284.0 → 168.1 284.0 → 140.2 289.1 → 173.2	45	18 30 18	Positive
HNE-MA HNE-MA (d3)	Lipid peroxidation	318.1 → 171.1 318.1 → 143.1 321.5 → 174.2	32	22 24 22	Negative
8-NO_2_Gua	Nitrative DNA damage	194.9 → 178.1 194.9 → 153.1	40	15 15	Negative
8-*Iso*-PGF_2β_	Lipid peroxidation	353.4 → 193.2 353.4 → 247.3	53	22 22	Negative

MassLynx 4.1 (Waters, UK) was used to control the LCMS system. TargetLynx (Waters, Manchester, UK) was used for data processing. The mass spectrometer was operated in the multiple reaction monitoring (MRM) mode. [M−H]^−^ and [M+H]^+^ were selected as molecular ions in ESI− and ESI+ respectively. MRM transitions and ESI parameters were obtained after direct infusion of each standard at a concentration of 100 ng mL^−1^ in the mass spectrometer. Optimised ESI parameters were as follows: capillary voltage 3.0 kV in ESI positive and 3.2 kV in ESI negative. The source temperature was 150 °C and the desolvation temperature was 400 °C. Nitrogen was used as nebulising and desolvation gas. The cone gas flow was 100 L h^−1^ and the desolvation gas flow was 550 L h^−1^. Argon was used as the collision gas. Optimised MS/MS parameters can be found in Table 1. Two MRM transitions, one for quantification and one for confirmation) were chosen for each compound. Only one MRM transition was selected for labelled internal standards.

The chosen methods were successful in the identification of 8-OHdG and achieved good separation of the negatively ionised biomarkers with elution at different retention times all within the first 10 min of the run.

**Fig. 2:**
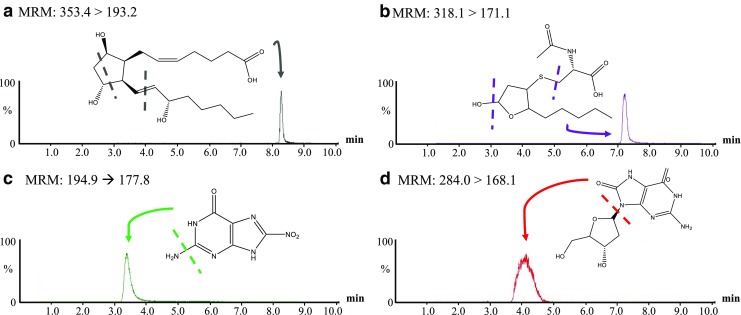
LC chromatograms and proposed structures of the quantifying mass fragment ions in mobile phase (80:20 H_2_O:MeOH). **a** 8-*iso*-PGF_2β_, m/z: 353.4 → 193.2. **b** HNE-MA, m/z: 318.1 → 171.1. **c** 8-NO_2_Gua, m/z: 194.9 → 177.8. **d** 8-OHdG, m/z: 284.0 → 168.1. Target analytes spiked at 500 μg L^−1^

Separation and identification of the quantifying mass fragment for each biomarker were successfully observed (Fig. 2). With regard to internal standards to allow quantification of target biomarkers in samples, a deuterated form of HNE-MA (HNE-MA-d3) was used for all three of the biomarkers ionised in negative mode. For 8-OHdG, a nitrogen labelled 8-OHdG (^15^N_5_-8-OHdG) was used as the internal standard in positive mode.

### Wastewater sample collection

Influent wastewater samples were collected via 24-h composite samples across a 7-day period from a wastewater treatment plant in the southwest of England serving a population equivalent ∼ 886,650. Once collected, samples were stored and transported upon ice to the laboratory for extraction and analysis. To account for both flows and population equivalent, daily loads per capita in mg/1000/people/day were calculated (Eq. 1).

1 $$ \mathrm{Daily}\ \mathrm{load}\ \mathrm{per}\ \mathrm{capita}=\mathrm{concentration}\times \mathrm{flow}\ \left({\mathrm{m}}^3\right)\times 1000\times \left(\frac{1000}{\mathrm{population}\ \mathrm{equivalent}}\right) $$

### Method validation

To establish regions of linearity, a triplicate injection of a 17-point calibration curve with concentrations ranging from 0.01–1000 μg L^−1^ was done for each compound. To determine inter- and intraday accuracy and precision, triplicate injections of three different concentrations were prepared within a 24-h period across three separate days. New solutions were made up for each separate day, the three different concentrations were 10 μg L^−1^ (50 μg L^−1^ for 8-OHdG), 100 μg L^−1^ and 500 μg L^−1^. Precision was calculated using relative standard deviation (RSD) for replicate injections (*n* = 6). Method inter- and intraday accuracy and precision were also established via the spiking of target biomarkers at initial concentrations of 0.5 μg L^−1^ and 1.25 μg L^−1^ into 100 mL of influent wastewater before the SPE step. Extracted samples were then injected in duplicate across a 24-h period and averages across the two concentrations were used to establish intraday method accuracy and precision (*n* = 3 injected in duplicate). To determine method interday accuracy and precision, fresh influent wastewater samples were prepared by spiking again at 0.5 μg L^−1^ and 1.25 μg L^−1^ and extracting before injecting as described above, across a further two 24-h periods.

Instrument detection limits (IDL) and instrument quantification limits (IQL) were established by the lowest concentrations which gave signal to noise ratios ≥ 3 and ≥ 10 respectively. The method detection limit (MDL) was calculated using the following:

2 $$ MDL=\frac{IDL\times 100}{Rec\times CF} $$

where IDL is the instrumental limit of detection, Rec is the relative SPE recovery of the analyte in wastewater and CF in the SPE concentration factor.

Method recoveries for each compound were determined by spiking of known amounts of analytes before extraction into 100 mL allotted influent wastewater samples at two different concentrations of individual analytes (0.5 μg L^−1^ and 1.25 μg L^−1^) with internal standards spiked into each sample at 100 ng and 500 ng for HNE-MA-d3 and ^15^N_5_-8-OHdG respectively. Method recoveries have been calculated as corrected recoveries (i.e. taking the internal standard concentration into consideration). This is calculated by the ratio of the concentration of target analytes in wastewater solutions when spiked before SPE (minus the concentration of analyte in the blank wastewater sample), divided by the standard mobile phase concentration (Eq. 3).

3 $$ {\mathrm{Method}\ \mathrm{recoveries}}_{\mathrm{corrected}}=\left(\frac{A_{\mathrm{spiked}\ \mathrm{before}\ \mathrm{SPE}}-{A}_{\mathrm{blank}}}{A_{\mathrm{mobile}\ \mathrm{phase}}}\right)\times 100\% $$

To determine matrix suppression, the ratio of the concentration of target analytes in wastewater samples spiked after SPE (minus the concentration of the analyte in the blank wastewater sample) is divided with the standard mobile phase sample concentration (Eq. 4).

4 $$ \mathrm{Matrix}\ \mathrm{supression}=\left(1-\frac{A_{\mathrm{spiked}\ \mathrm{after}\ \mathrm{SPE}}-{A}_{\mathrm{blank}}}{A_{\mathrm{mobile}\ \mathrm{phase}}}\right)\times 100\% $$

### Biomarker stability in wastewater

To assess the stability of the target analytes within wastewater, a 24-h wastewater stability study was performed. A total of four 2 L reactors of influent wastewater was used, two of which were kept at 17 °C with the other two kept at 4 °C to determine if any degradation occurred at two different temperatures. Each reactor was spiked with each target analyte to determine a final concentration of 250 μg L^−1^. After initial analyte spiking, 2 × 50 mL samples were taken from each reactor and spiked with respective internal standards before filtering and SPE extraction to give concentration at time 0. After which, a further five sampling points were taken across the 24 h (0, 2, 4, 6, 12, 24 h) with 2 × 50 mL samples taken from each reactor at the time point. For calculating average concentration of target analytes at each sampling point, the average of both the two samples was taken at each time point along with the average across duplicate reactors. Errors were calculated using the standard deviation of concentrations across duplicate reactors and duplicate samples (*n* = 4).

## Results and discussion

### Method validation

#### LC-MS validation parameters

Regarding the calibration curves, the mean coefficients of determination (*R*^2^) gave excellent linearity with values ≥ 0.997 for all four biomarkers over the concentration range investigated (0–500 μg L^−1^ or 0–1000 μg L^−1^) (Table 2). However, not all biomarkers displayed acceptable linearity (*R*^2^ ≥ 0.997) across the entire concentration range studied. 8-NO_2_Gua required two calibration curves, 0.1–100 μg L^−1^ and 100–1000 μg L^−1^ at *R*^2^ at 0.998 and 0.999 respectively. Intra- and interday accuracy exhibited acceptable ranges of 94–107% for all biomarkers. Regarding intra- and interday precision, all four biomarkers displayed very small deviations giving > 97% for all biomarkers investigated.

**Table 2:** Instrument performance data detailing linearity including instrument detection limits (IDLs) and instrument quantification limits (IQLs) and intra- and interday accuracy and precision for all biomarkers studied

Compound	Internal standard	Linearity		IDL (μg L^−1^)	IQL (μg L^−1^)	Intraday instrument performance		Interday instrument performance	
		Range (μg L^−1^)	*R* ^2^			Accuracy (%)	Precision (%)	Accuracy (%)	Precision (%)
8-OHdG	^15^N_5_-8-OHdG	5–500	0.997	1	5	95.6	97.7	97.1	97.5
HNE-MA	HNE-MA-d3	0.5–1000	0.999	0.01	0.5	103	98.4	106	98.1
8-NO_2_Gua	HNE-MA-d3	0.1–100 100–1000	0.998 0.997	0.01	0.1	107	97.7	94.1	97.3
8-*Iso*-PGF_2β_	HNE-MA-d3	0.5–1000	0.999	0.05	0.5	99.2	97.8	101	98.8

Instrument detection limits (IDL) were as low as 0.01 μg L^−1^ for both HNE-MA and 8-NO_2_Gua and 0.05 μg L^−1^ for 8-*iso*-PGF_2β_. Instrument quantification limits (IQLs) were generally low at < 0.5 μg L^−1^. 8-OHdG displayed slightly poorer sensitivities at 1 μg L^−1^ for detection and 5 μg L^−1^ for quantification.

#### SPE-LC-MS validation parameters

Regarding method sensitivity within wastewater matrices, method detection limits (MDLs) of < 0.2 ng L^−1^ were achieved for HNE-MA, 8-*iso*-PGF_2β_ and 8-NO_2_Gua. HNE-MA in particular gave excellent method sensitivity with an MDL at 0.0590 ng L^−1^. Method quantification limits (MQLs) for the same three biomarkers were also < 3 ng L^−1^. 8-OHdG on the other hand gave slightly poorer method sensitivity (17.1 ng L^−1^ and 85.3 ng L^−1^ for MDL and MQL respectively), Results from method recoveries are all reported as corrected recoveries (i.e. the internal standards have been considered). HNE-MA gave excellent recoveries with minimal matrix suppression across the two concentrations studied (85% and 17% averages respectively over 0.5 and 1.25 μg L^−1^). Signal enhancement was observed for 8-*iso*-PGF_2β_ (− 67% and − 55% at 0.5 and 1.25 μg L^−1^ respectively) and high method recoveries were exhibited (142% and 147% at 0.5 and 1.25 μg L^−1^ respectively). This is attributed to the challenges in identifying 8-*iso*-PGF_2β_ amongst the peaks it occurs in within the unspiked wastewater sample. With regard to 8-OHdG and 8-NO_2_Gua, both had lower but reproducible method recoveries (average recoveries of 32% and 65% respectively across 0.5 and 1.25 μg L^−1^). Furthermore, both compounds were moderately susceptible to a wastewater matrix, with 8-OHdG average of 47% signal suppression and 8-NO_2_Gua at 46% across 0.5 and 1.25 μg L^−1^. Regarding method accuracy, HNE-MA had excellent method accuracy results across the 3 days studied with 101% for interday accuracy (Table 3). The higher but consistent method accuracies observed for 8-*iso*-PGF_2β_ at 140% and 134% for intra- and interday are attributed to again being unable to identify the biomarker peak in the unspiked wastewater samples. Therefore concentrations of this biomarker already present in real wastewater samples were not accounted for in calculations. 8-NO_2_Gua exhibited acceptable method accuracies at 88.9% for interday whilst 8-OHdG displayed low method accuracies at 64.1% for interday. The lower method accuracies observed for 8-OHdG is a reflection of the challenges of analysing this biomarker in real wastewater samples, further evidenced by the results of matrix effects and method recoveries (Table 3). Regarding method precision, both inter- and intraday precision gave acceptable ranges of 90–96% for all four biomarkers studied.

**Table 3:** Method performance data detailing method detection limits (MDLs) and method quantification limits (MQLs), method recoveries and matrix effects, intra- and interday accuracy and precision for all biomarkers studied (*n* = 3 injected in duplicate)

Compound	Method linearity (ng L^−1^)	MDL (ng L^−1^)	MQL (ng L^−1^)	Method recoveries (%)		Matrix effects (%)		Intraday method performance		Interday method performance	
				0.5 (μg L^−1^)	1.25 (μg L^−1^)	0.5 (μg L^−1^)	1.25 (μg L^−1^)	Accuracy (%)	Precision (%)	Accuracy (%)	Precision (%)
HNE-MA	3.0–5903	0.06	3.0	83.4	86.0	18.0	15.9	91.2	95.1	101	95.6
8-*Iso*-PGF_2β_	1.7–3455	0.17	1.7	142	147	− 67.2	− 55.2	140	95.2	134	94.4
8-NO_2_Gua	1.3–13,123	0.13	1.3	67.9	61.3	48.6	44.2	86.5	90.4	88.9	90.2
8-OHdG	85.3–8532	17.1	85.3	29.0	35.1	53.0	40.5	63.2	91.8	64.1	92.5

### 8-*Iso*-PGF_2β_ and its isomers

**Fig. 3:**
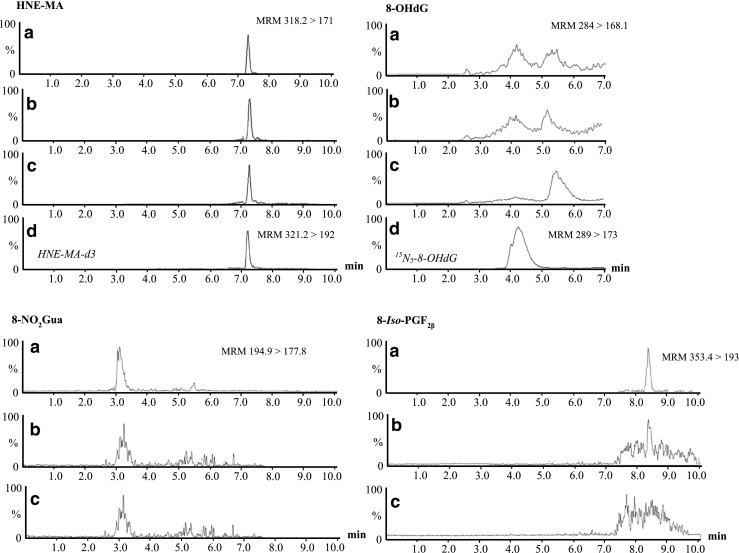
LC chromatograms of the quantifying mass fragment ions of each target biomarker in influent wastewater samples. (a) Initial spiked analyte concentration of 0.5 μg L^−1^, (b) initial spiked analyte concentration of 0.5 μg L^−1^, (c) unspiked wastewater, (d) internal standards: HNE-MA-d3 (spiked at 100 ng L^−1^) or ^15^N_5_-8-OHdG (spiked at 500 ng L^−1^)

Interestingly, when studying 8-*iso*-PGF_2β_ in wastewater, instead of a clearly resolved peak that is observed within the mobile phase, there is a broad, poorly resolved series of peaks eluting between 6 and 10 min in wastewater. However, when spiked with the target analyte at initial concentrations of 0.5 μg L^−1^ and 1.25 μg L^−1^, 8-*iso*-PGF_2β_ can be identified amongst the series of peaks (Fig. 3). A possible explanation for this observation could be due to the presence of a wide range of F2-isoprostane compounds in wastewater. 8-*iso*-PGF_2β_ belongs to a large family of prostaglandin-like isomers known as the isoprostanes. The isoprostanes are metabolic products of the peroxidation of the arachidonic acid via a free radical catalysed mechanism [31]. Fatty acids like arachidonic acid occur with relative abundance in human cells and are crucial components as they facilitate the proper formation and function of cell membranes. From the peroxidation of arachidonic acid, four classes of F2-isoprostanes may be formed [26]. The F2-isoprostane regioisomer compromises of eight diastereoisomers that arise to 64 different F2-isoprostanes. So if 8-*iso*-PGF_2β_ is present in wastewater, then it is highly likely the other isomers excreted in urine will also be present. SPE is widely recognised as a non-specific extraction technique, and with the combination of Oasis HLB cartridges will ultimately result in the extraction of a wide number of compounds including those with similar chemistries. Furthermore, such similar isomers are likely to have matching mass fragment peaks hence the potential elution of different fragment ions around the target analytes potentially resulting in the interference observed.

It should be noted that it is recognised in the literature of the lack of clarity in whether a number of analytical methods for F2-IsoPs in biological matrices are specific for a single isomer or whether it is capturing numerous isobaric species [36–38]. For example, Davies et al. demonstrated various dinor, dihydro F2-IsoP metabolite species being captured within a single chromatographic peak in urine samples via tandem LC-MS techniques [39]. Due to the significant number of various stereo- and regioisomers of the F2-IsoP family, the analytical challenges of separation and reliability of peaks given are well recognised within biological matrices such as urine. It is unsurprising therefore that such difficulties are similarly observed within more complex matrices such as wastewater. However, such challenges have been overcome in WBE, as previously mentioned, Ryu et al. used highly selective immunoassay techniques to capture 8-*iso-*PGF_2α_ from wastewater to give a single isomer species [29].

However, it is important to study the isoPs as a group in WBE, particularly as it is not currently known which isomer indicates oxidative stress the best or even which isomer is most abundant in urine. This idea was partially explored in an extensive review by Daughton reviewing the potential of isoPs for use in WBE, in particular, it was highlighted that F2t-IsoPs including 8-*iso*-PGF_2α_ was one of the first ones to became widely available to purchase; hence, much of the early studies are based upon this [12]. Indeed, it has been widely agreed that the study of isoPs as a marker of oxidative stress in clinical studies should be studied as a group and metabolites should also be included [40–42]. This would not only reduce complications of variability of excretion amounts thereby improving reliability, but by capturing and studying the F2-isoprostanes could help in creating a standardised analytical method for use both in clinical fields and WBE. Further work is currently undertaken by the authors to identify and quantify all relevant F2-isoprostanes.

### Stability of biomarkers in wastewater

**Fig. 4:**
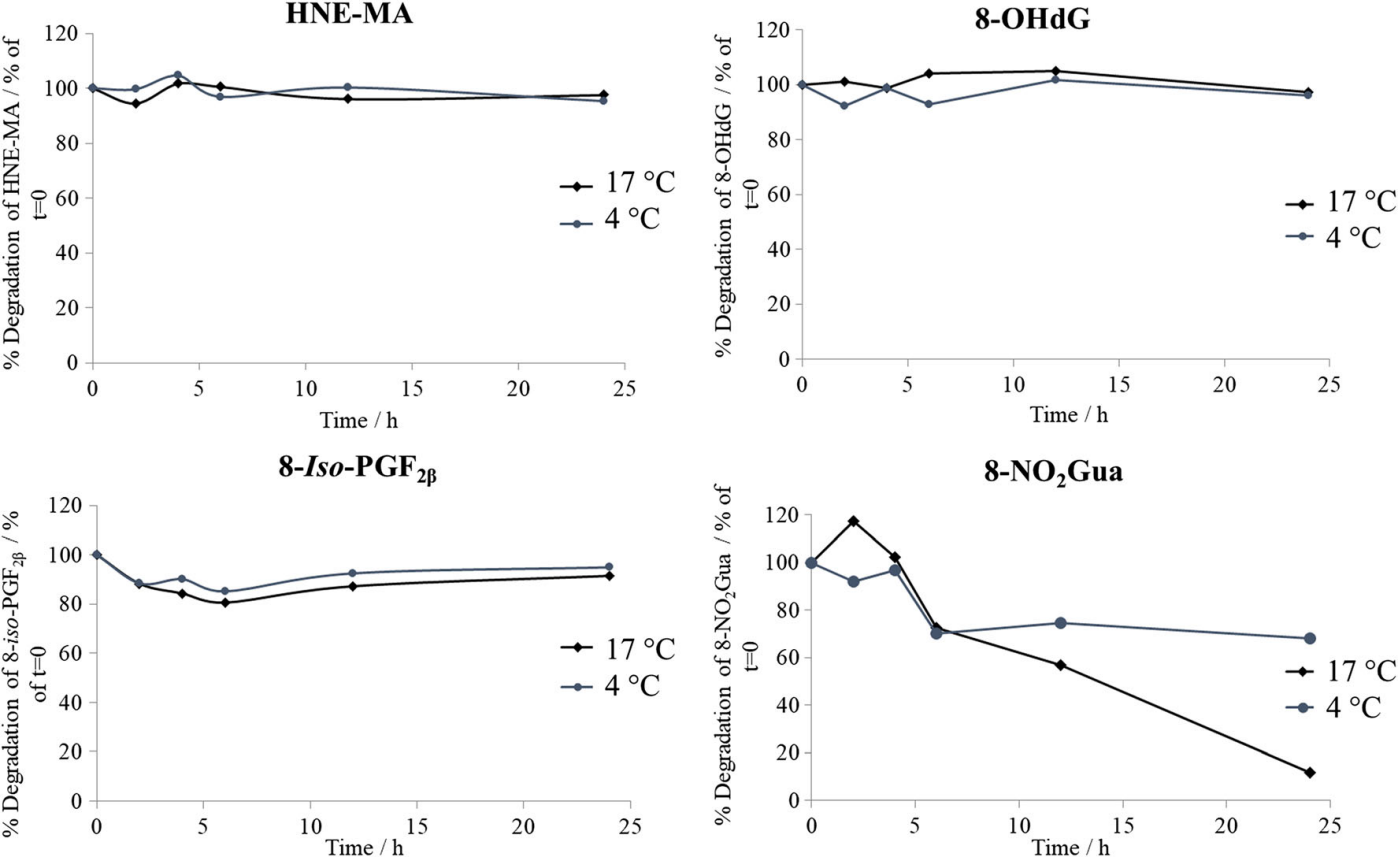
Stability of each target biomarker in influent wastewater incubated at 17 °C and 4 °C (*n* = 4) over 24 h. Initial analyte spiking of 1.25 μg L^−1^ within each 2 L reactor (final concentration in 500 μL at *t* = 0, 250 μg L^−1^)

Whilst the behaviours of target biomarkers in clinical matrices, for example in urine, are well reported [28], the stability and presence of such compounds have not been previously reported in wastewater (with the exception of 8-*iso*-PGF_2α_). Results from the 24-h wastewater biomarker stability study displayed positive results for the majority of the biomarkers studied (Fig. 4). HNE-MA, 8-OHdG and 8-*iso*-PGF_2β_ all demonstrated high stabilities with little degradation at both 4 °C and 17 °C in wastewater over the 24-h period (> 90% of the concentration spiked at *t* 0 h remaining at time period 24 h). Interestingly for 8-NO_2_Gua, significant degradation was observed over the 24-h period with both reactors at 4 °C and 17 °C with 68% and 10% of the concentration spiked at *t* 0 h remaining at 24 h respectively. This indicates that 8-NO_2_Gua, however useful as a biomarker, might show low stability in wastewater. Conversely, initial screening of the biomarkers in wastewater has shown 8-NO_2_Gua to be successfully detected and quantifiable at a concentration of 0.0832 ± 0.519 μg L^−1^, *n* = 3 (Fig. 3).

### Wastewater analysis

**Fig. 5:**
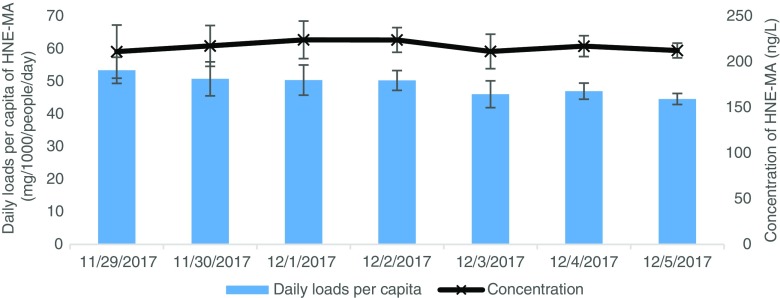
Daily loads per capita and concentration of HNE-MA in influent wastewater over period of 29 November 2017–05 November 2017. 24-h composite samples used with errors calculated by standard deviation (*n* = 4)

When spiked into wastewater, all four biomarkers were detected and quantified at their characteristic retention times of 7.45, 3.5, 8.22 and 4.11 min for HNE-MA, 8-NO_2_Gua, 8-*iso*-PGF_2β_ and 8-OHdG respectively (Fig. 3). To further test the validated method, a sampling campaign compromising of 24-h composite influent wastewater samples were studied over 7 days. As markers of oxidative stress and lipid peroxidation, it was assumed that daily loads of target analytes would not experience significant weekly variations and should give relatively stable concentrations across the sampling period. Results demonstrated that HNE-MA gave excellent resolved peaks on all days of the campaign and could be quantified every day. Using influent flowrates and the population of the WWTP, daily loads per capita of HNE-MA were calculated (Fig. 5). Observed levels of HNE-MA averaged at 48.9 ± 4.07 mg/1000/people/day across 7 days sampled. 8-*iso-*PGF_2β_ was detected with good intensity but was found amongst the broad series of peaks as previously mentioned. 8-OHdG was detected, albeit at < LOQ. 8-NO_2_Gua on the other hand was not detected on any of the 7 days investigated; as previously mentioned, this might be attributed to its low stability within wastewater samples. A potential factor of why this biomarker was observed in previous screening of wastewater samples could be dilution (e.g. wetter weather causing variable flows). This shall be addressed in future work to verify the results, in particular due to the stability of biomarker investigation into whether grab sample over composite samples would be more appropriate.

## Conclusion

To conclude, we have reported, for the first time, the development of an analytical method using SPE and UHPLC-MS/MS techniques for the detection and quantification of four biomarkers of oxidative stress in wastewater. The method showed very good performance: accuracy (> 87%), precision (> 90%), method quantification limits (1.3–3.0 ng L^−1^) and biomarker stability in wastewater for HNE-MA, 8-OHdG and 8-*iso-*PGF_2β_. In contrast, 8-NO_2_Gua was found to be less stable in wastewater (68% and 10% of the concentration spiked at *t* 0 h remaining at 24 h respectively at 4 °C and 17 °C), which affected its method performance: accuracy (> 63%), precision (> 91%), method quantification limits (85.3 ng L^−1^). All four biomarkers were detected within wastewater samples but full quantification of only HNE-MA was carried out. HNE-MA was quantified in wastewater at levels averaging at 48.9 ± 4.1 mg/1000/people/day. 8-*iso-*PGF_2β_ was detected within the broad series of peaks as previously mentioned; further work is required in order to investigate separation. 8-OHdG was detected, albeit at < MQL due to relatively low MQL for this biomarker. To the authors’ knowledge, HNE-MA has never been observed and quantified successfully in wastewater before. Further work is required to fully evaluate suitability of 8-NO_2_Gua as a biomarker due to its low stability. More extensive sample preparation utilising sorbents of higher selectivity and higher concentration factor should be also considered to allow for full quantification of 8-OHdG and 8-*iso-*PGF_2β_.
